# Biosynthetic Studies on Water-Soluble Derivative 5c (DTX5c)

**DOI:** 10.3390/md10102234

**Published:** 2012-10-12

**Authors:** Tamara S. Vilches, Manuel Norte, Antonio Hernández Daranas, José J. Fernández

**Affiliations:** 1 University Institute for Bio-Organic Chemistry “Antonio González”, University of La Laguna, Av. Astrofísico Francisco Sánchez 2, La Laguna 38206, Spain; Email: tvilches@ull.es (T.S.V.); mnorte@ull.es (M.N.); 2 Department of Organic Chemistry, Faculty of Pharmacy, University of La Laguna, Av. Astrofísico Francisco Sánchez s/n, La Laguna 38206, Spain; 3 Department of Chemical Engineering and Pharmaceutical Technology, Faculty of Pharmacy, University of La Laguna, Av. Astrofísico Francisco Sánchez s/n, La Laguna 38206, Spain

**Keywords:** biosynthesis, marine toxin, DSP, marine polyether, polyketide

## Abstract

The dinoflagellate *Prorocentrum belizeanum *is responsible for the production of several toxins involved in the red tide phenomenon known as Diarrhetic Shellfish Poisoning (DSP). In this paper we report on the biosynthetic origin of an okadaic acid water-soluble ester derivative, DTX5c, on the basis of the spectroscopical analysis of ^13^C enriched samples obtained by addition of labelled sodium [l-^13^C], [2-^13^C] acetate to artificial cultures of this dinoflagellate.

## 1. Introduction

One of the main points of interrelation between chemical research on marine natural products and seafood quality control has been oriented towards chemical, structural and biosynthetic studies of secondary metabolites responsible for a variety of toxic syndromes. Within these intoxications, the toxic phenomenon known as Diarrhetic Shellfish Poisoning (DSP) occurs with frequency in northwest Europe, Canada and Japan, generating great alarm from the public health point of view and in turn within the shellfish industry. The main clinical symptoms of these intoxications consist of diarrhea, vomiting and headache, which are a result of the ingestion of shellfish contaminated by okadaic acid (OA) and dinophysistoxins (DTXs) by feeding on planktonic organisms. In addition, the scientific community has shown an enormous interest in these phenomena due to the complex structures of the toxins involved and the potent biological activities characteristic of these substances [1,2,3].

In general, the toxins involved are OA and its congeners, which include several okadaates, lipophilic dinophysistoxins (DTX1–3) and the water-soluble derivatives (DTX4 and DTX5a–c) [3]. As regards their bioactivity, these molecules stand as potent tumour promoters as well as potent and highly selective inhibitors of protein phosphatases type 1 (PP1) and 2A (PP2A), making them extremely useful tools for studying cellular processes regulated by phosphorylation, such as transduction, or cell division and memory [4,5,6]. From a structural point of view, all these toxins are polyethers with a 38 carbon-atom backbone and six or seven pendant methyl groups. They are characterized by the presence of oxolane and oxane rings, which form spiro or trans-fused systems. Despite their enormous structural diversity, these polyketide metabolites are related by their common origin from highly functionalized carbon chains whose assembly is controlled by multifunctional enzyme complexes, the polyketide synthases (PKSs). Each condensation is followed by a cycle of modifying reactions: ketoreduction, dehydration and enoyl reduction. In contrast to the fatty acid biosynthesis in which reductive modifications normally follows each condensation, the PKSs can use this sequence in a highly selective and controlled manner to assemble polyketide intermediates with an enormous number of permutations in functionality along the chain. These peculiarities have encouraged intensive biosynthetic studies to determine the biosynthetic pathways of these biotoxins group [7,8,9,10]. Thus, the biosynthetic origin of the carbon backbone of DTX1 and OA was first determined and subsequently continued by the study of okadaates and water-soluble toxins DTX4, DTX5a and DTX5b [11,12,13,14,15].

**Figure 1:**
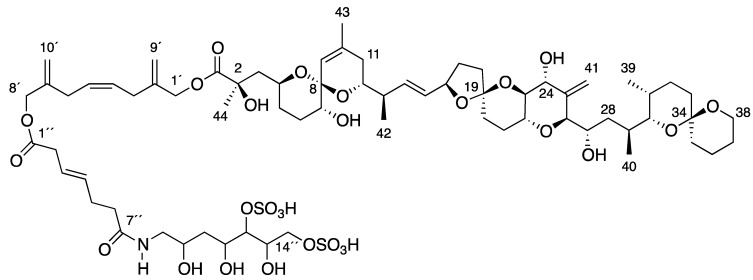
Structure of Water-Soluble Derivative 5c (DTX5c).

Here we report on the biosynthetic origin of the most recently determined water-soluble toxin DTX5c (Figure 1) [16,17] by addition of stable isotopic precursors to a culture of the dinoflagellate *Prorocentrum belizeanum*.

## 2. Results and Discussion

DSP toxins are produced in small quantities by dinoflagellates. Therefore, optimization of the culturing conditions is needed in order to maximize yield. This in turn, necessitates the development of an easy and efficient purification method. All the procedures must also be applicable to cultures involved in the feeding with labeled precursors. 

**Figure 2:**
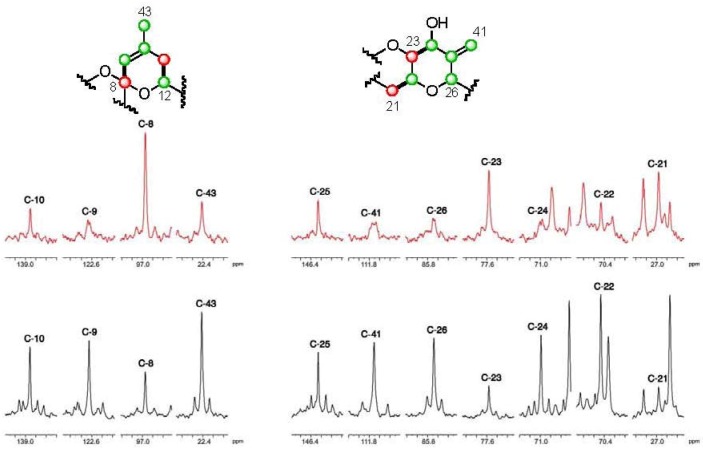
Selected sections of ^13^C NMR spectra in CD_3_OD for the fragment C-8–C-10–C-43 and C-21–[C-41]–C-26 in labeled samples of DTX5c. In red, those corresponding to a sample obtained after addition of [1-^13^C] sodium acetate [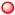], and in black those after addition of [2-^13^C] sodium acetate experiment [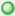].

The toxin studied in this work, DTX5c, was produced by an axenic culture of the dinoflagellate *Prorocentrum belizeanum *[16,17]. Previous biosynthetic studies undertaken with similar metabolites indicated that only basic metabolic precursors could be successfully used with species of *Prorocentrum *[15]. In consequence, our biosynthetic study of DTX5c was designed as a series of feeding experiments using labeled [1-^13^C] and [2-^13^C] sodium acetate as the metabolic precursors that were added to artificial cultures of *P. belizeanum *(PBMAO1 strain) (Figure 2). A typical feeding experiment starts with inoculation of dinoflagellates into seven 5L Erlenmeyer flasks containing 3 L of Guillard K medium. Cells were grown for four days and at that point a mixture of two antibiotics, penicillin (40 IU/mL) and streptomycin sulphate (200 IU/mL) were added into the media. One day later, the labeled precursor was added up to a final concentration of 0.67 mM [12]. Subsequently, three weeks later, the culture was harvested. Finally, the cells were filtered and extracted with MeOH. The extract was chromatographed using Sephadex LH-20, followed by reverse phase (C-18) chromatography and the final purification was carried out by HPLC on a XTerra column, yielding approximately 0.3 mg of DTX5c (15 μg/L) in each experiment.

**Figure 3:**
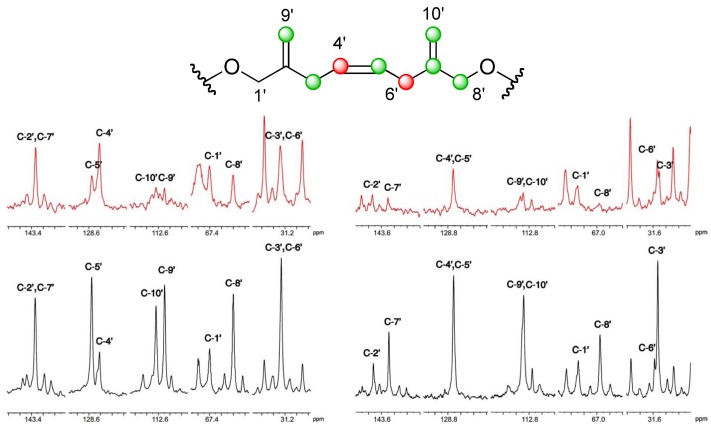
Selected sections of the ^13^C NMR spectra of the C-1′–C-10′ fragment using CD_3_OD (left) and using pyridine-*d*_5_ (right) as solvents. In red, those corresponding to a sample obtained from the [1-^13^C] sodium acetate experiment [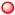], and in black those from the [2-^13^C] sodium acetate experiment [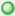].

**Figure 4:**
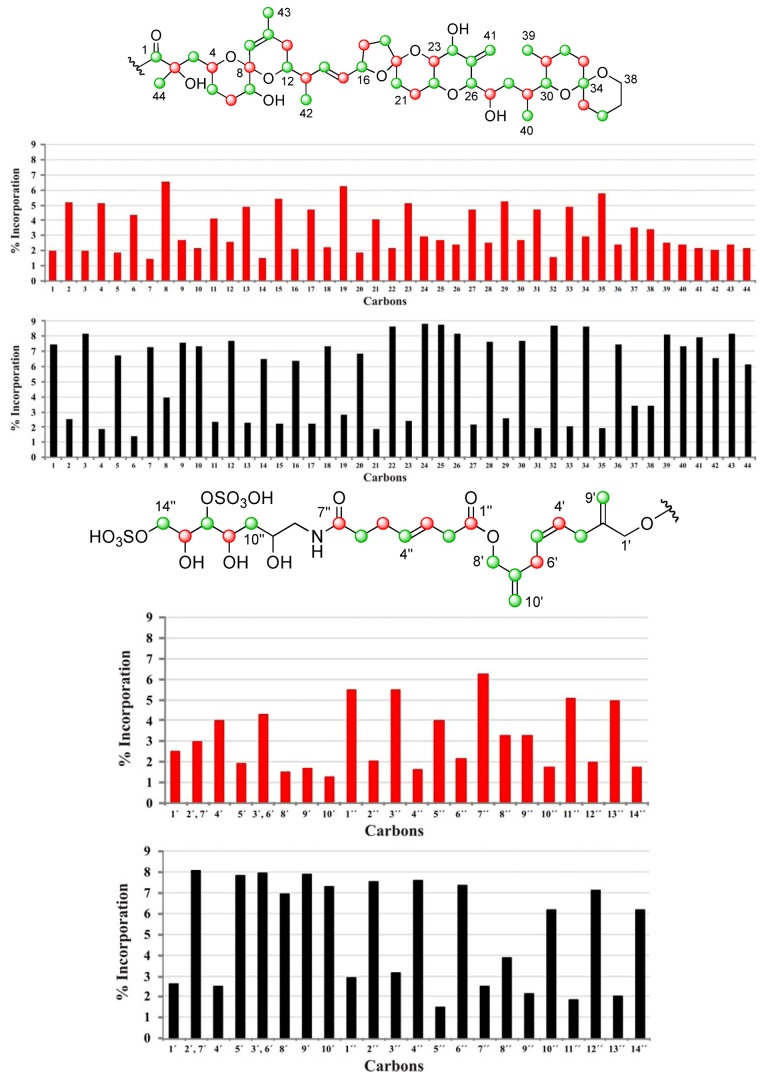
Labeling pattern observed in ^13^C enrichment experiments for DTX5c. [1-^13^C] sodium acetate experiment is represented by [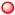], and the [2-^13^C] sodium acetate experiment is represented by [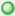].

The results obtained from the different biosynthetic experiments revealed different degrees of incorporation at all positions except for six carbon atoms located at C-37, C-38, C-1′, C-2′, C-8″ and C-9″ where no distinctive enrichment was observed using sodium acetate. Quantitative measurements indicated that ^13^C enrichment was to sufficient to continue the analysis, (total av. ^13^C 7.62% ± 0.8 SD in [2-^13^C] sodium acetate experiment) although the specificity was smaller than that obtained in previous studies performed on *Prorocentrum lima* cultures. Nevertheless, comparison of the ^13^C-NMR spectra obtained from addition of different labeled precursors showed the complementarity between the enrichment patterns, as could be observed in Figure 2, Figure 3,Figure 4 and Table 1. In fact, enrichment of 24 carbons upon addition of [1-^13^C] sodium acetate and 38 carbons using [2-^13^C] sodium acetate confirmed that 62 out of the 68 carbons in DTX5c derive from acetate, including all the pendant methyl groups. Furthermore, upon addition of [2-^13^C] sodium acetate, it was clearly observed that, a number of signals showed the characteristic splitting due to ^13^C–^13^C spin-spin coupling arising from the existence of adjacent ^13^C enriched positions that is characteristic of some marine compounds [15]. As was already reported, slightly higher than average enrichments were observed at C-37 and C-38, however the biosynthetic origin of these carbons have been described to be deriving from glycolate [11,13].

As regards the OA moiety (C-1→C-44) of DTX5c, we found the expected incorporation pattern [11,12]. Thus, two alternative fragments interrupted the classic polyketide profile. The first one was found at C-9–C-10–C-43 and was detected by the existence of two weak signals that flanked C-9 and C-43, whereas the carbon signal from C-10 was flanked by four signals, with constant coupling values (*J*_9–10_ = 72.1 Hz and *J*_10–43_ = 42.8 Hz). The second exception was observed within the C-24→C-26 moiety. This time the coupling constants values between C-24–C-25 and C-25–C-26 were very similar (*J*_24–25_ = 42.6 Hz; *J*_25–26_ = 42.5 Hz), while C-25–C-41 showed a *J*_25–41_ = 73.7 Hz. (Figure 2). 

**Table 1:** ^13^C NMR data for DTX5c in CD_3_OD (300 K; 150 MHz).

C	δ_C_	Origin	% Inc a [2-^13^C]	% Inc a [1-^13^C]	C	δC	Origin	% Inc a [2-^13^C]	% Inc a [1-^13^C]
**1**	176.05	m	7.5	2.0	**34**	96.21	m	8.6	2.9
**2**	74.93	c	2.5	5.1	**35**	36.21	c	1.9	5.7
**3**	45.39	m	8.2	2.0	**36**	19.01	m	7.5	2.4
**4**	67.57	c	1.9	5.1	**37**	25.73		3.4	3.5
**5**	32.62	m	6.7	1.8	**38**	60.56		3.4	3.4
**6**	27.21	c	1.4	4.3	**39**	10.27	m	8.1	2.5
**7**	72.26	m	7.3	1.4	**40**	15.89	m	7.4	2.4
**8**	96.86	c	4.0	6.6	**41**	111.70	m	7.9	2.2
**9**	122.61	m	7.6	2.7	**42**	15.79	m	6.6	2.1
**10**	138.87	m	7.3	2.2	**43**	22.41	m	8.2	2.4
**11**	33.19	c	2.3	4.1	**44**	25.34	m	6.1	2.2
**12**	71.46	m	7.7	2.6	**1′**	67.38		2.6	2.5
**13**	42.20	c	2.3	4.9	**2′, 7′**	143.35		8.0	3.0
**14**	135.78	m	6.5	1.5	**4′**	128.42	c	2.5	4.0
**15**	131.58	c	2.2	5.4	**5′**	128.56	m	7.8	1.9
**16**	79.64	m	6.4	2.1	**3′, 6′**	31.23		7.9	4.3
**17**	30.86	c	2.2	4.7	**8′**	66.97	m	6.9	1.5
**18**	37.24	m	7.3	2.2	**9′**	112.45	m	7.9	1.7
**19**	106.34	c	2.8	6.3	**10′**	112.60	m	7.3	1.3
**20**	33.36	m	6.9	1.9	**1″**	172.47	c	2.9	5.5
**21**	26.92	c	1.9	4.0	**2″**	37.96	m	7.5	2.0
**22**	70.42	m	8.7	2.2	**3″**	123.29	c	3.1	5.5
**23**	77.56	c	2.4	5.1	**4″**	133.38	m	7.5	1.6
**24**	70.97	m	8.8	2.9	**5″**	29.01	c	1.4	4.0
**25**	146.25	m	8.7	2.7	**6″**	35.88	m	7.3	2.2
**26**	85.66	m	8.2	2.4	**7″**	174.99	c	2.5	6.3
**27**	65.27	c	2.2	4.7	**8″**	45.67		3.8	3.2
**28**	35.97	m	7.6	2.5	**9″**	68.43		2.1	3.2
**29**	31.51	c	2.6	5.2	**10″**	38.01	m	6.2	1.8
**30**	76.02	m	7.7	2.7	**11″**	69.06	c	1.8	5.1
**31**	27.94	c	1.9	4.7	**12″**	79.82	m	7.1	2.0
**32**	26.70	m	8.7	1.5	**13″**	70.76	c	2.0	4.9
**33**	30.46	c	2.1	4.9	**14″**	70.27	m	6.2	1.7

^a^ ^13^C quantification was made using CH_2_Cl_2_ as internal standard.

**Figure 5:**
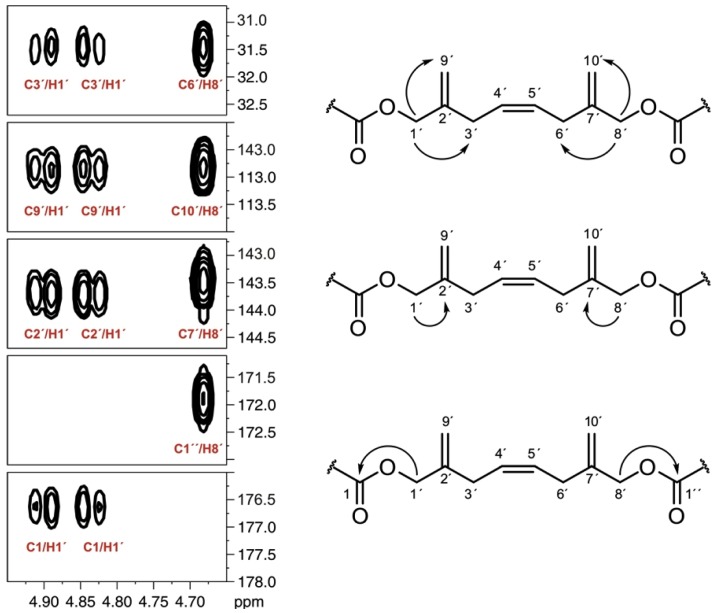
Significant HMBC correlations for fragment C1′→C10′ in DTX5c using pyridine-*d*_5_.

At first sight, the isotopic labeling pattern observed along the ester side chain of DTX5c was also similar to that reported for other related water-soluble toxins such as DTX5a and DTX5b [14], but our observations were hindered by the structural symmetry observed in the C1′→C10′ fragment. Thus, we faced an overlapping problem with the ^13^C NMR signals for two pairs of carbons at C-2′, C-7′ and C-3′, C-6′ when using CD_3_OD as solvent (Figure 3). Consequently, it was impossible, under these conditions, to unambiguously assure their biogenetic origin. This problem was resolved by the alternative use of pyridine-*d*_5_ as solvent, which shows a complementary chemical shifts profile (Figure 5).

As a result the previously mentioned pairs of carbons C-2′–C-7′ and C-3′–C-6′ were now distinguishable while C-9′–C-10′ and C-4′–C-5′ were overlapped. Combining both spectra the assignment was completed and it was clear that ^13^C enrichment occurred at C-3′, C-5′, C-7′, C-8′, C-9′ and C-10′ upon addition of [2-^13^C] sodium acetate while only C-4′ and C-6′ were enriched when [1-^13^C] sodium acetate was used (Table 2). Finally, within this fragment, C-1′ and C-2′ did not incorporate ^13^C distinctly from sodium acetate. 

**Table 2:** ^13^C NMR data for the C-1′→C-10′ fragment in DTX5c (300 K; 150 MHz).

Carbon	δ_C_ CD_3_OD	δ_C_ Pyridine-*d*_5_	% Inc * [2-^13^C] a	% Inc * [1-^13^C] a
**1** **′**	67.38	67.28	2.6 ^b^	2.5 ^b^
**2** **′**	143.35	143.73	1.9 ^c^	1.8 ^c^
**3** **′**	31.23	31.53	6.6 ^c^	1.5 ^c^
**4** **′**	128.42	128.72	2.5 ^b^	4.0 ^b^
**5** **′**	128.56	128.72	7.8 ^b^	1.9 ^b^
**6** **′**	31.23	31.56	1.3 ^c^	2.8 ^c^
**7** **′**	143.35	143.50	6.1 ^c^	1.2 ^c^
**8** **′**	66.97	66.94	6.9 ^b^	1.5 ^b^
**9** **′**	112.45	112.84	7.9 ^b^	1.7 ^b^
**10** **′**	112.60	112.84	7.3 ^b^	1.3 ^b^

^a^ ^13^C quantification was made using CH_2_Cl_2_ as internal standard; ^b^ Using CD_3_OD; (c) Using pyridine-*d*_5_.

**Figure 6:**
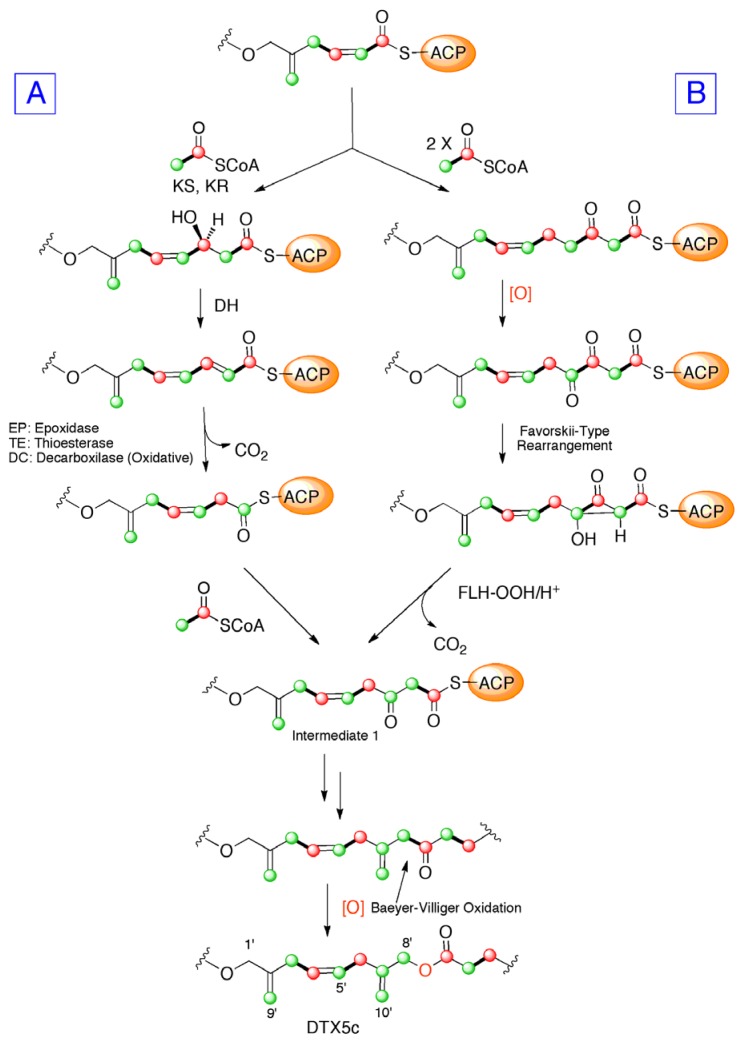
Biosynthesis of DTX5c according to (**A**) Shimizu’s or (**B**) Wright’s proposals. Enrichment from [1-^13^C] or [2-^13^C] sodium acetate experiments are represented by [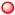] and [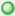], respectively.

Moreover ^13^C signals of C-7′, C-8′, and C-10′, appeared flanked by “satellite” signals when cultures were fed with [2-^13^C] sodium acetate. Coupling constant values for these carbons, *J*_C7__′__–C10′_ = 73.6 Hz and *J*_C7__′__–C8′_ = 46.5 Hz, were consistent with the carbon hybridization. These data are in concordance with the existence of a “(**m**)-**m**-**m**” sequence, similar to others previously observed in other polyketide isolated from dinoflagellates. The truncation of the carbon chain in marine polyethers to give vicinal “**m**-**m**” systems in the polyketide precursor has been explained in several ways. The initial proposal, which includes a biochemical process involving dicarboxylic building blocks derived from the TCA cycle [10,11,15], was discarded and a second more plausible explanation given by Wright and co-workers was published. This proposal was based on the occurrence of a Favorskii or a benzyl-benzylic rearrangement in the carbon chain that would move the connectivity to the next carbon to give rise to the hypothetical intermediate 1 (Figure 6, pathway B) [13,14]. The last hypothesis was published by Shimizu (Figure 6, pathway A) and involved a terminal α,β-unsaturated acid (crotonic acid type) in the nascent polyketide chain. This would undertake epoxidation and the resultant α,β-epoxy carboxylic acid undergo facile decarboxylation [18]. The later two proposals explains the appearance of **m**-**m** systems in an intermediate position of the carbon chain, as well as the fact that oxidation to a carbonyl is necessary to incorporate the pendant methyl group (C-10′), following a process similar to that suggested in the irregular moieties of okadaic acid. However, in our opinion the proposal made by Shimizu explains better the biosynthetically counterintuitive appearance of a methyl-derived aldehyde group at the terminal carbons of brevetoxins as well as the carboxylic group of okadaic acid (both enriched in feeding experiments with 2-^13^C sodium acetate). 

Thus, in accordance with the above comments, the ^13^C the incorporation pattern observed in the C-1′→C-10′ side chain of DTX5c, could be explained by the assembly of four intact acetate units to a glycolate-starting unit (C-1′–C-2′) [14]. At this point, the necessary β-keto thioester—where the C-7′ carbonyl group derives from [2-^13^C] sodium acetate—would be generated. Next, an acetate unit would attack this carbonyl. The polyketide chain would continue its growth and should be divided after a Baeyer-Villiger oxidation in a “**m**-**c**” bond (Figure 6). Nevertheless, the real pathway used by dinoflagellates to achieve these particular rearrangements is still a challenge for those marine natural products research groups interested in biosynthetic studies.

## 3. Experimental Section

### 3.1. Instrumentation and General Methods

NMR spectra were recorded on a Bruker AVANCE III 600 instrument equipped with a 5 mm TCI cryoprobe. NMR spectra were obtained dissolving DTX5c in CD_3_OD (99.8 + atom% D, Aldrich, USA) and pyridine-*d*_5_ (HDO + D_2_O < 0.05%; 99.5 atom% D, Eurisotop). Chemical shifts are reported relative to TMS (δ_H_ 0.0 ppm) at 300 K and coupling constants were calculated in Hz. NMR assignments were obtained from examination of 1D and 2D experiments (^1^H, ^13^C, COSY, HSQC, and HMBC). Spectral widths of 7200 and 34,500 Hz and acquisition times of 2.27 and 0.47 s were used in ^1^H and ^13^C experiments respectively. ^13^C experiments were acquired using the inverse gated decoupling pulse sequence. 30° pulses and relaxation delays of 30 s and 5 s were used in the ^1^H and ^13^C NMR experiments respectively in order to ensure quantitative measurements. Prior to Fourier transformation, zero filling was performed to expand the data to at least double the number of acquired data points. Exponential window functions with line broadening coefficients ranging from 0.1 to 3 Hz were used. HPLC analyses were performed on a Waters instrument equipped with a differential diffractometer detector and an X-Terra column. TLC’s were carried out using Si gel Merck 60G, and were visualized with 10% phosphomolybdic acid in ethanol.

### 3.2. *Prorocentrum belizeanum* Cultures

The strain of the dinoflagellate *P. belizeanum* used in this work (PBMA01), originally isolated from a coral reef of La Reunion Island, Indian Ocean, France, was obtained from the culture collection of phytoplankton cultures at the Centro Oceanográfico at Vigo, by courtesy of Santiago Fraga. Cultures of *P. belizeanum* were grown in 250 mL flasks containing 150 mL of sea water enriched with Guillard K medium at 23 °C, at a salinity of 35, with an irradiance of 60 μE s^−1^ m^−2 ^and under a 18:6 light:darkness photo cycle. Cultures were incubated statically for 6 weeks up to a final volume of 1.5 L.

### 3.3. Preparation of *Prorocentrum belizeanum* Isotopic Enriched Cultures

Feeding experiments involved inoculation of a 21 L Guillard K medium, divided in seven portions, with 3 L of dinoflagellate culture with constant white fluorescent illumination. These 21 L of culture were incubated for 4 days at which time the antibiotics penicillin (40 IU/mL) and streptomycin sulphate (200 IU/mL) were added. On day 4, the labeled precursor at a final concentration of 0.67 mM, was added. The culture was then harvested two weeks after addition of the labeled precursor.

### 3.4. Extraction and Isolation of DTX5c Labelled Samples

The cells from labeled cultures were filtered and extracted with MeOH. The extract was chromatographed on Sephadex LH 20 using methanol as eluent. The fractions that containing the enriched toxin were chromatographed on reverse phase C-18 and the final purification was carried out in a HPLC Water instrument using a XTerra column eluted whit MeOH:Water 4:1, and around 0.3 mg of DTX5c was isolated and characterized in each experiment. 

## 4. Conclusions

The biosynthetic origin of DTX5c produced by the dinoflagellate *P. belizeanum* is consistent with those reported from other related metabolites isolated from different species (*P. lima* and *P. maculosum*). Addition of ^13^C enriched metabolic precursors into cultures of the dinoflagellate allowed us to track the incorporation of intact acetate units within the toxin. However, the polyketide backbone of DTX5c is interrupted by three unusual “**m**-**m**” sequences that have only been observed in natural marine products. Thus, the most significant novelty between the previously reported derivatives and DTX5c is the existence of an unusual fragment “(**m**)-**m**-**m**” in the ester side chain. The biogenesis of this sequence can be rationalized either by epoxidation of the carbon backbone resulting in a α,β-epoxy carboxylic acid that undergo facile decarboxylation (Figure 6A) or by elimination of one carboxyl-derived carbon of acetate via a Favorskii-type reaction (Figure 6B). The incorporation of the pendant methyl carbon would take place by addition to the previously oxidized methyl derived carbon. Finally, a Baeyer-Villiger oxidation would insert an oxygen atom within the carbon backbone at 14 carbon atoms of distance from the end terminus of the polyketide chain, as has been observed in other DTX toxins produced by the genus *Prorocentrum*.
